# The Potential of Genetics in Identifying Women at Lower Risk of Breast Cancer

**DOI:** 10.1001/jamaoncol.2023.5468

**Published:** 2023-12-28

**Authors:** Alexandre Bolze, Elizabeth T. Cirulli, Catherine Hajek, Jamie M. Schnell Blitstein, Joseph J. Grzymski

**Affiliations:** 1 Helix, San Mateo, California; 2 Renown Health, Reno, Nevada; 3 University of Nevada, Reno

## Abstract

**Importance:**

Genetic information is not being used to identify women at lower risk of breast cancer or other diseases in clinical practice. With the new US Preventive Services Task Force guidelines lowering the age for mammogram screening for all, there is a potential benefit in identifying women at lower risk of disease who may defer the start of mammographic screening. This genetic risk-based approach would help mitigate overscreening, associated costs, and anxiety.

**Objective:**

To assess breast cancer incidence and age of onset among women at low genetic risk compared with women at average risk and evaluate the potential to delay mammography on the basis of genetic risk stratification.

**Design, Setting, and Participants:**

This retrospective case-control study included 25 591 women from the Healthy Nevada Project sequenced by Helix between 2018 and 2022. Data extracted from electronic health records at the end of 2022 (mean length of electronic health record available was 12 years) were used for the analysis in 2023.

**Main Outcomes and Measures:**

Breast cancer diagnosis was identified from electronic health records. Classification to the low-risk genetic group required (1) the absence of pathogenic variants or a variant of uncertain significance in *BRCA1*, *BRCA2*, *PALB2*, *ATM,* or *CHEK2*, and (2) a low polygenic risk score (bottom 10%) using a 313–single-nucleotide variant model.

**Results:**

Of 25 591 women in the study (mean [SD] age was 53.8 [16.9] years), 2338 women (9.1%) were classified as having low risk for breast cancer; 410 women (1.6%) were classified as high risk; and 22 843 women (89.3%) as average risk. There was a significant reduction in breast cancer diagnosis among the low-risk group (hazard ratio, 0.39; 95% CI, 0.29-0.52; *P* < .001). By 45 years of age, 0.69% of women in the average-risk group were diagnosed with breast cancer, whereas women in the low-risk group reached this rate at 51 years. By 50 years of age, 1.41% of those in the average-risk group were diagnosed with breast cancer, whereas those in the low-risk group reached this rate at age 58 years. These findings suggest that deferring mammogram screening by 5 to 10 years for women at low risk of breast cancer aligns with new draft recommendations.

**Conclusions and Relevance:**

The findings of this retrospective case-control study underscore the value of genetics in individualizing the onset of breast cancer screening. Improving breast cancer risk stratification by implementing both high-risk and low-risk strategies in screening can refine preventive measures and optimize health care resource allocation.

## Key Points

**Question:**

Can genetic information identify women for whom it is safe to delay mammogram screening?

**Findings:**

In this case-control study of 25 591 women, 2338 (9.1%) were classified as having low genetic risk for breast cancer; these women exhibited significantly later onset of breast cancer compared with average-risk or high-risk counterparts, indicating a potential to defer mammogram screening by 5 to 10 years.

**Meaning:**

Delaying the age to start mammogram screenings for women at low genetic risk could optimize health care resource allocation.

## Teaser

This case-control study assesses breast cancer incidence among women at low genetic risk compared with women at average risk, evaluating the potential to delay mammography on the basis of genetic risk stratification.

## Introduction

In May 2023, the US Preventive Services Task Force (USPSTF) recommended biennial mammogram screening for all women aged 40 to 74 years to detect early-stage cancer.^1^ This recommendation entails screening an additional 20 million women.^2^ Although earlier screening will benefit many, it raises concerns about overscreening and its implications, prompting us to investigate whether we could identify women at lower risk of breast cancer who might defer mammograms. Guidelines exist for identifying women at high risk of breast cancer,^3,4,5^ but there are no guidelines for those at low risk. Early-onset breast cancers often arise from rare germline pathogenic variants (P variants), which is why genetics, such as the presence of a germline P variant in *BRCA1* or *BRCA2 *genes, is used to identify women at high risk.^3,5,6^ We hypothesized that genetics could also identify a subset of women at decreased risk. To test this hypothesis, women were classified as low risk if they met both criteria: (1) absence of P variants and variants of uncertain significance (VUS) in breast cancer genes (*BRCA1*, *BRCA2*, *PALB2*, *ATM*, and *CHEK2*),^3,6,7,8,9^ and (2) having a low (bottom 10%) polygenic risk score (PRS) using a validated 313–single-nucleotide variants (SNVs) model.^10^ Using these criteria, we compared breast cancer incidence among those considered at low risk with their average-risk counterparts.

## Methods

In this case-control study, we performed a retrospective analysis of 25 591 women from the all-comers Healthy Nevada Project,^8,11^ who had available electronic health records from Renown Health (mean length of electronic health records, 15 years; eTable 1 in Supplement 2). The University of Nevada, Reno, institutional review board approved the study (project 956068-12). All participants provided written informed consent between 2018 and 2022.

Breast cancer diagnoses were determined from electronic health records using the *International Statistical Classification of Diseases, Tenth Revision, Clinical Modification (ICD-10-CM)* codes C50, D05, and Z85.3. Of the total participants, 1295 women (5.1%) had a diagnosis of breast cancer at the end of 2022.

Genetic data were obtained through the Helix Exome assay, which combines clinical-grade exome sequencing with a microarray-equivalent backbone sequencing,^11^ enabling imputation of common SNVs for PRS calculation.^8^ Participants were assigned a PRS percentile based on their rank within the genetic similarity group, ensuring no exclusion due to genetically inferred ancestry (eMethods in Supplement 1).

## Results

**Figure 1.**
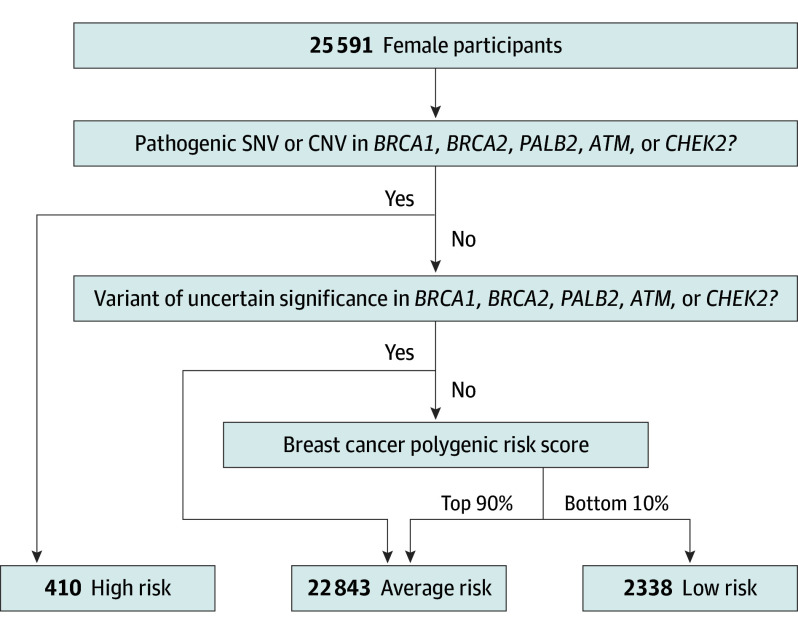
Genetic Risk Stratification of Female Participants Schema detailing how genetic risk stratification was done. Detailed methods about Exome+ sequencing, and variant annotation with VEP, ClinVar, and LOFTEE based on the MANE transcripts for each gene can be found in the eMethods in Supplement 1. CNV indicates copy number variant; SNV, single nucleotide variant.

**Figure 2.**
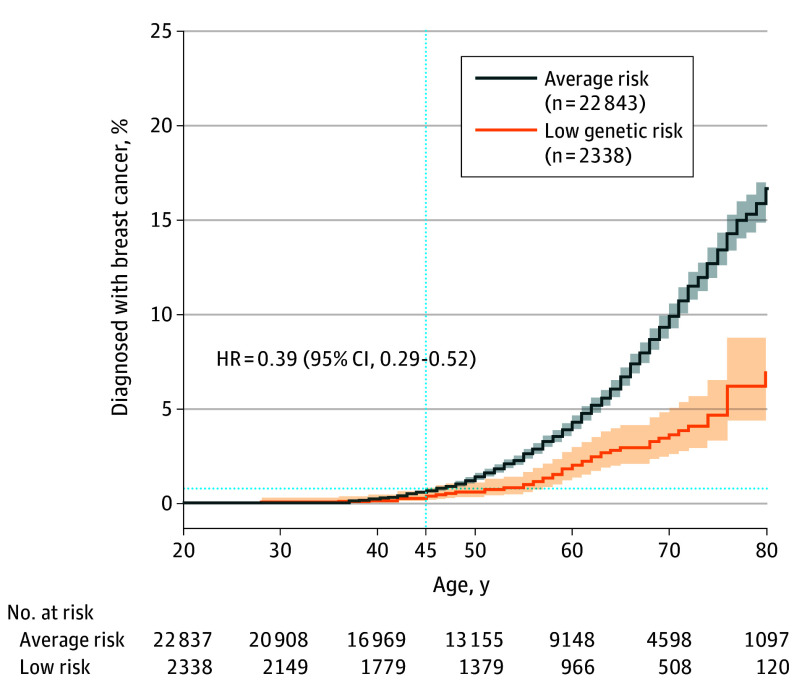
Percentage of Women With Breast Cancer Diagnosis by Age and Genetic Risk Group Kaplan-Meier curves showing the percentage of women with a breast cancer diagnosis by age based on their genetic risk group (average risk, blue-gray curve; low risk, orange curve). The blue dashed line indicates the percentage of women with a breast cancer diagnosis by age 45 years. The 95% CIs are represented in light blue-gray and light orange shading. The Kaplan-Meier Fitter function (to draw the curves) and the CoxPHFitter function (to calculate the hazard ratio [HR]) were used and were from the Lifelines Python library.

Of 25 591 women in the study (mean [SD] age, 53.8 [16.9] years), 410 (1.6%) were classified as high risk with a P variant in *BRCA1*, *BRCA2*, *PALB2*, *ATM*, or *CHEK2*. An additional 1967 women had a VUS in these genes (eFigure 1 in Supplement 1, eTables 2-4 in Supplement 2). Women with a P variant had a significantly increased risk compared with those without a P variant or a VUS (hazard ratio [HR], 5.7; 95% CI, 4.7-7.1; log-rank *P* < .001) (eFigure 2 in Supplement 1). However, women with a VUS had no increased risk (HR, 1.1; 95% CI, 0.9-1.3; *P* = .53) (eFigure 2 in Supplement 1). Although these results showed that most VUS had no significant association with disease risk, historical data from ClinVar have shown that some VUS are reclassified as pathogenic.^12^ Therefore, women with a VUS were excluded from the low-risk group (Figure 1). Next, the 313-SNVs PRS was associated with breast cancer diagnosis in the study cohort (eTable 5 in Supplement 2). Women in the bottom 10% of the PRS distribution had a decreased risk of breast cancer compared with those with an average PRS (between 41% and 60%; HR, 0.48; 95% CI, 0.36-0.65; *P* < .001) (eFigure 3 in Supplement 1). Overall, 410 women (1.6%) were classified as high risk (presence of a P variant), 22 843 women (89.3%) as average risk, and 2338 women (9.1%) as low risk (no P or VUS variant and a PRS in the bottom 10%) (Figure 1). There were significantly fewer breast cancer diagnoses in the low-risk category compared with those at average risk (HR, 0.39; 95% CI, 0.29-0.52; *P* < .001) (Figure 2).

To assess the potential to defer screening, the cumulative risk of breast cancer was calculated at 5 and 10 years after the recommended start of biennial mammography. By the age of 45 years (5 years after the recommended age to start mammogram screening), 0.69% of women at average risk had been diagnosed with breast cancer, a rate not reached by women in the low-risk group until the age of 51 years (Figure 2; Table). We also tested whether expanding the number of genes analyzed would detect any of the 10 women in the low-risk group diagnosed with breast cancer by age 50 (Figure 2; Table). Whether any of the 10 women in the low-risk group diagnosed with breast cancer by age 50 years were detected was tested by expanding the number of genes analyzed. However, only 48 women of 25 591 (0.2%) had a P variant in 1 of these 6 additional genes (*BARD1, CDH1, MAP3K1, RAD51C, RAD51D*, or *TP53*; eFigure 1 in Supplement 1). A total of 38 women in the previously defined low-risk group were reclassified—4 carried a P variant and 34 a VUS in the *BARD1*, *CDH1*, *MAP3K1*, *RAD51C*, *RAD51D,* or *TP53* gene—none of whom were diagnosed with breast cancer at the time of this study. Overall, the results remained unchanged (eFigure 4 in Supplement 1, eTable 6 in Supplement 2), indicating that deferring mammogram screening by 5 to 10 years for women in the low-risk group would lead to a similar screening performance compared with the current USPSTF guidelines.

**Table.** Cumulative Risk of Breast Cancer Diagnosis at Different Ages

Variable	Genetic risk group,^a^ No. (%)		Hazard ratio (95% CI)^b^
	Average	Low	
No.	22 843 (89.3)	2338 (9.1)	NA
With breast cancer by age 35 y	17 (0.08)	1 (0.04)	0.57 (0.08-4.30)
With breast cancer by age 40 y	55 (0.30)	3 (0.15)	0.66 (0.21-2.10)
With breast cancer by age 45 y	117 (0.69)	7 (0.40)	0.53 (0.25-1.13)
With breast cancer by age 50 y	218 (1.41)	10 (0.60)	0.48 (0.26-0.91)
With breast cancer by age 55 y	365 (2.62)	15 (0.99)	0.40 (0.24-0.66)
With breast cancer by age 60 y	537 (4.30)	26 (2.03)	0.47 (0.32-0.70)
With breast cancer by age 65 y	735 (6.70)	34 (2.95)	0.46 (0.32-0.64)
With breast cancer by age 70 y	929 (9.90)	38 (3.65)	0.40 (0.29-0.55)

Abbreviation: NA, not applicable.

^a^ A total of 410 women (1.6%) were classified as having a high risk of breast cancer (presence of a pathogenic variant).

^b^ The reference group was patients at average risk.

## Discussion

Current screening guidelines do not adequately account for interindividual variability in breast cancer risk,^1,13^ and when they aim to account for interindividual variability, they specifically focus on identifying those at higher risk.^3,4^ The findings of this retrospective case-control study suggest that rare and common variants can also be combined to identify women at lower risk of breast cancer. These findings also validate this stratification in a large, unselected cohort that measured breast cancer incidence. PRS is particularly useful to define a lower-risk group because rare variants were only present in less than 10% of individuals (of 25 591 women, 410 had a P variant [1.6%], and 1967 had a VUS [7.7%]), and rare variants alone cannot separate those with average risk from those with lower risk. These results indicate that women at low genetic risk have a similar risk of breast cancer at age 51 years as those at average risk at age 45 years, and a similar risk at age 58 years as those at average risk at age 50 years. Based on the US Census Bureau there are approximately 14 million women in the US aged 40 to 47 years,^2^ indicating that approximately 1.3 million women would be at low risk using the genetic approach detailed in this study. This genetic screening strategy could potentially avoid 650 000 mammograms each year under the new USPSTF guidelines.^1^

### Limitations

The low genetic risk classification strategy used in this study can potentially be improved. Alternate methods that encompass a broader gene set and exclusively rely on P variants without considering VUS may yield different results. In addition, although the polygenic risk model used is well-validated,^5,10^ new models are expected to improve polygenic risk prediction. Efforts are underway to develop (1) models with higher accuracy for diverse populations,^14^ and (2) disease subtype–specific models.^15^ Lastly, women with a family history of breast cancer may still be at higher risk, despite having a low risk based on the genetic information measured.

## Conclusions

The results of this retrospective case-control study underscore the value of genetics in reducing overscreening, associated overdiagnosis, costs, and anxiety by identifying patients at low risk of breast cancer who may be able to defer mammogram screening to later in life. Implementing recommendations to decrease screening based on low-risk factors may pose challenges for physicians, but reliable risk assessment is essential to support informed decisions and build patient trust in both high-risk and low-risk scenarios. Using a validated genetic risk stratification tool could reduce guideline discrepancy and improve clinician efficacy in shared decision-making discussions regarding individualized screening plans.

## Notes

Supplement 1. **eMethods.** Supplementary Methods **eFigure 1.** Classification of Pathogenic Variants and Variants of Uncertain Significance **eFigure 2.** Impact of Pathogenic Variants and VUSs on Breast Cancer Diagnosis **eFigure 3.** Impact of Polygenic Risk on Breast Cancer Diagnosis **eFigure 4.** Impact of Low Genetic Risk Based on 11 Genes and a 313-SNVs PRS on Breast Cancer Diagnosis

Supplement 2. **eTable 1.** Description of the Healthy Nevada Project **eTable 2.** List of All Pathogenic Variants in 11 Breast Cancer Genes **eTable 3.** List of All Pathogenic CNVs in 11 Breast Cancer Genes **eTable 4.** List of all VUSs in 11 Breast Cancer Genes **eTable 5.** Association of the 313-SNVs Breast Cancer PRS and Breast Cancer in the Healthy Nevada Project Cohort **eTable 6.** Impact of Delay of Screening for Those in the Low-Risk Group Defined Using 11 Genes and a PRS **eReferences.** Supplemental References

Supplement 3.
Data Sharing Statement
