# Supplementary material for: The Potential of Genetics in Identifying Women at Lower Risk of Breast Cancer
Source: JAMA Oncol. 2023 Dec 28;10(2):236–9. doi: 10.1001/jamaoncol.2023.5468 (PMC10870185; doi:10.1001/jamaoncol.2023.5468)
Supplement: Supplement 1. — eMethods. Supplementary Methods eFigure 1. Classification of Pathogenic Variants and Variants of Uncertain Significance eFigure 2. Impact of Pathogenic Variants and VUSs on Breast Cancer Diagnosis eFigure 3. Impact of Polygenic Risk on Breast Cancer Diagnosis eFigure 4. Impact of Low Genetic Risk Based on 11 Genes and a 313-SNVs PRS on Breast Cancer Diagnosis [file jamaoncol-e235468-s001.pdf]

Supplemental Online Content

Bolze A, Cirulli ET, Hajek C, Blitstein JS, Grzymski JJ. The potential of genetics in identifying women at lower risk of breast cancer. *JAMA Oncol.* Published online December 28, 2023. doi:10.1001/jamaoncol.2023.5468

Supplement 1. eMethods. Supplementary Methods

eFigure 1. Classification of Pathogenic Variants and Variants of Uncertain Significance

eFigure 2. Impact of Pathogenic Variants and VUSs on Breast Cancer Diagnosis

eFigure 3. Impact of Polygenic Risk on Breast Cancer Diagnosis

eFigure 4. Impact of Low Genetic Risk Based on 11 Genes and a 313-SNPs PRS on Breast Cancer Diagnosis

Supplement 2. eTable 1. Description of the Healthy Nevada Project

eTable 2. List of All Pathogenic Variants in 11 Breast Cancer Genes

eTable 3. List of All Pathogenic CNVs in 11 Breast Cancer Genes

eTable 4. List of all VUSs in 11 Breast Cancer Genes

eTable 5. Association of the 313-SNPs Breast Cancer PRS and Breast Cancer in the Healthy Nevada Project Cohort

eTable 6. Impact of Delay of Screening for Those in the Low-Risk Group Defined Using 11 Genes and a PRS

eReferences. Supplemental References

This supplemental material has been provided by the authors to give readers additional information about their work.

© 2023 American Medical Association. All rights reserved.

Supplementary Methods

Participants

This study was based on the Healthy Nevada Project. The Healthy Nevada Project study was reviewed and approved by the University of Nevada, Reno Institutional Review Board (IRB, project 956068-12), and all participants provided informed consent. The initial dataset comprised 39,546 individuals. For this study, we only included participants who were inferred to be of female sex based on the genetic data and had longitudinal data length >0 in the electronic health records at Renown Health.

Clinical phenotypes from EHR

All participants included in this study had Electronic Health Records available from Renown Health. Phenotypes were processed from Epic/Clarity EHR data. This study is centered on breast cancer, and we did not look at ovarian cancer despite the known impact of pathogenic variants in *BRCA1* and *BRCA2* on both breast and ovarian cancers. We also did not look at breast cancer subtypes (ER+ / ER-, PR+ / PR- or triple negative), or any other types of cancer.

Exome+® sequencing

DNA was extracted from saliva. All samples were sequenced at Helix using the Exome+® assay. The Exome+® assay targets the exome as well as about 300,000 common SNPs outside of the exome that allow for imputation of all common SNPs genome-wide. In turn, this allows GWAS analysis and polygenic risk score calculation. Data was processed using a custom version of Sentieon and aligned to GRCh38, with variant calling and phasing algorithms following GATK best practices. Imputation of common variants in the HNP data was performed by pre-phasing samples and then imputing.

© 2023 American Medical Association. All rights reserved.

Variant annotation and classification

Variant annotation was performed with Ensembl Variant Effect Predictor-99[16] and LOFTEE[17]. The MANE transcripts were used to determine variant consequences[18]. ClinVar annotations were based on the file: clinvar_20220723.vcf.gz downloaded from https://ftp.ncbi.nlm.nih.gov/pub/clinvar/vcf_GRCh38/ .

We first selected a set of five genes: *BRCA1*, *BRCA2*, *PALB2*, *ATM* and *CHEK2* for our study. These are the genes with the strongest association with breast cancer risk[3,8,19,20]. Genes such as *BARD1*, *RAD51C* or *RAD51D* have also been associated with breast cancer and have also been used in studies developing models to predict breast cancer risk in the population[5]. Other genes such as *TP53* or *CDH1* are involved in cancer syndromes, and protein-truncating variants in these genes predispose to cancer, including breast cancer, with a high penetrance[21,22]. Lastly, protein truncating variants in *MAP3K1*[7] have recently been associated with breast cancer in the largest exome-wide study of breast cancer to date. This is why we included these six genes (*BARD1*, *CDH1*, *MAP3K1*, *RAD51C*, *RAD51D* and *TP53*) in a supplementary analysis. The list of genes could be expanded further and more studies will be necessary to identify the optimal set of genes to analyze when the goal is to identify women at lower risk of breast cancer.

We classified variants based on the decision tree shown in **Figure S1**. All of the variants classified as ‘Pathogenic’ are listed in **Table S2**. This includes information on the number of carriers for each variant, as well as the mean sequencing depth for each variant. All variants classified as a variant of uncertain significance (VUS) are reported in **Table S4**.

This method led to 2,362 women (9.2%) with a VUS variant in one of these genes. We took the conservative approach to exclude these individuals from the low-risk category. Future studies will be necessary to define the best way to classify rare variants that lead to exclusion of a carrier from the low-risk group. The name ‘VUS’ may not be appropriate either.

© 2023 American Medical Association. All rights reserved.

CNV annotation and classification

CNVs that passed quality control thresholds were annotated with overlapping MANE transcripts. For this study, Copy number of 1 (instead of 2) or 0 were considered pathogenic. We did not consider CNVs for VUS. Specifically, we did not consider a copy number of 3 or more as a VUS.

Polygenic risk score calculation

We used the 313-SNPs PRS model published in 2019[10]. Full details on how we implemented this score and its performance in the Healthy Nevada Project cohort can be found in our paper regarding higher risk in women with a pathogenic variant in *ATM* and *CHEK2* and a high polygenic risk[8]. We reported that this PRS model achieved an AUC of 0.63 in participants in the Europe genetic similarity group and an AUC of 0.66 in the Americas genetic similarity group[8], which was consistent with the original publication of this PRS[10].

In a nutshell, we first converted the model from GRCh37 to GRCh38. We then calculated the score in each of the 25,591 women included in this study. Our cohort is diverse and the distribution across genetic similarity (inferred from genetic data) was the following: N Africa = 499; N Americas = 3,728; N East Asia = 832; N Europe = 19,484; N Other = 929 and N South Asia = 119. There are known challenges in implementing one model across a diverse cohort, which is why we took the following steps. First, a genotype dosage was calculated for each variant in the score for each individual. The dosage was based on the genotype probability field resulting from the imputation pipeline. When an individual had no GP or no GT (genotype) for a specific variant, the dosage was based on the Allele Frequency of this variant in gnomAD v3 for the population closest to the genetic similarity of the participant. We then split the cohort into 6 cohorts based on genetic similarity and ranked individuals based on their PRS value. The distribution of the PRS values was slightly shifted for different groups. The median PRS was lowest in the Europe population, and highest in the East Asia population. To reduce biases, we

© 2023 American Medical Association. All rights reserved.

therefore assigned a percentile based on the ranking within the participant's genetic similarity distribution. Lastly, we regrouped all six genetic similarity groups into one cohort for later analyses that were based on percentiles.

Survival analysis, hazard ratios and statistical tests

Kaplan Meier survival curves were done using the KaplanMeierFitter function from the Lifelines python library. Statistical differences between survival curves were assessed using a logrank_test function from the lifelines.statistics python library.

Values at a given age (e.g. 45 years old, 5 years after the recommended age to start screening) were calculated using the 'predict' function.

Hazard Ratios were calculated using the CoxPHFitter function from the lifelines python library.

Plots were made using pyplot from the matplotlib python library.

© 2023 American Medical Association. All rights reserved.

Supplementary Figures

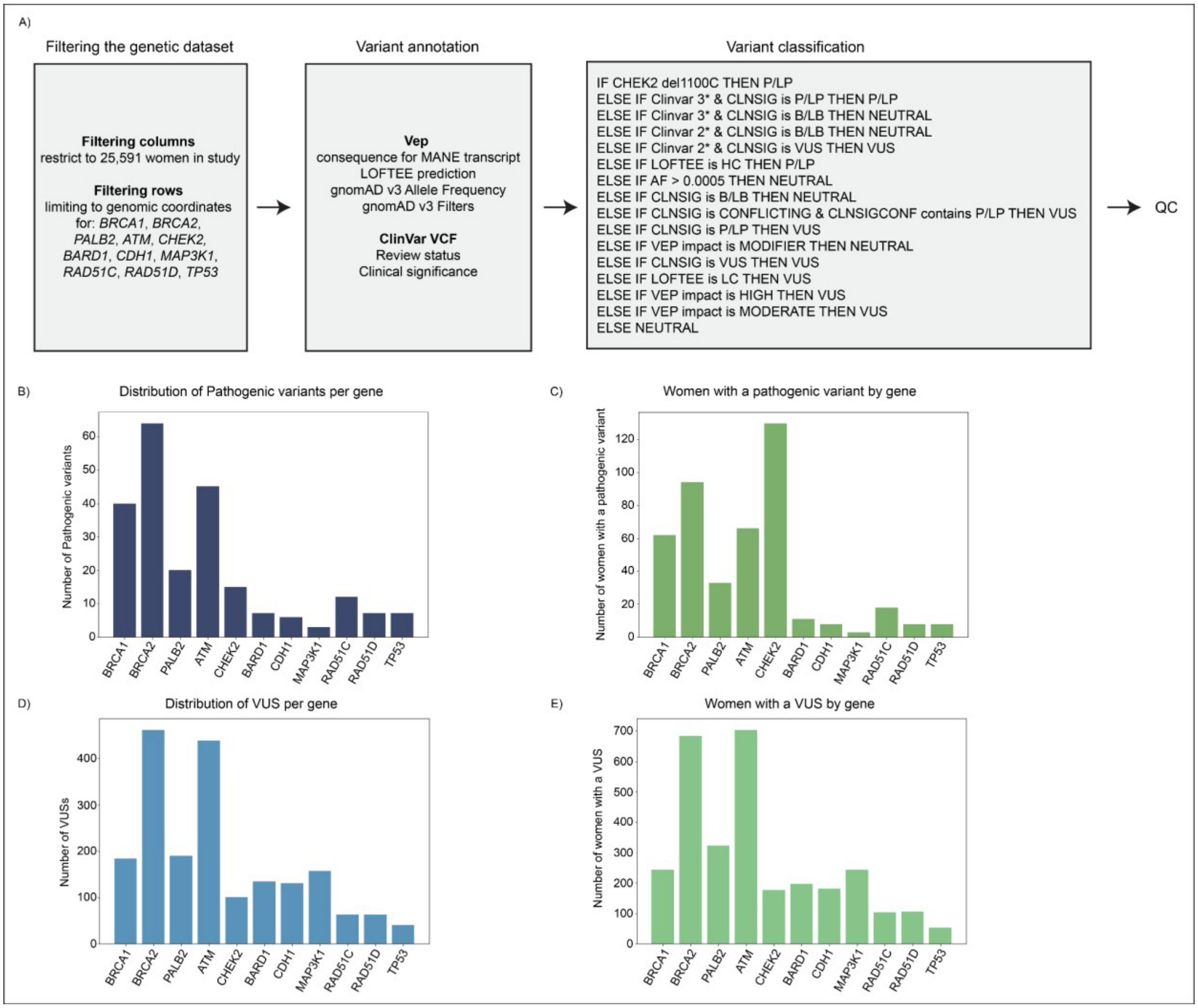

eFigure 1: Classification of pathogenic variants and variants of uncertain significance

(A) Schematic of the workflow for variant annotation and classification. Clinvar 3* represents a Clinvar review status of ‘Reviewed by Clingen expert panel’. Clinvar 2* represents a Clinvar review status of ‘Multiple submitters, no conflicts’. **(B)** Number of unique pathogenic variants identified by gene. List of all variants in **Table S2**. **(C)** Number of women carrying a pathogenic variant by gene. All variants were heterozygous. **(D)** Number of unique variants of uncertain significance (VUS) identified by gene. List of all variants in **Table S4**. **(E)** Number of women carrying a VUS by gene.

© 2023 American Medical Association. All rights reserved.

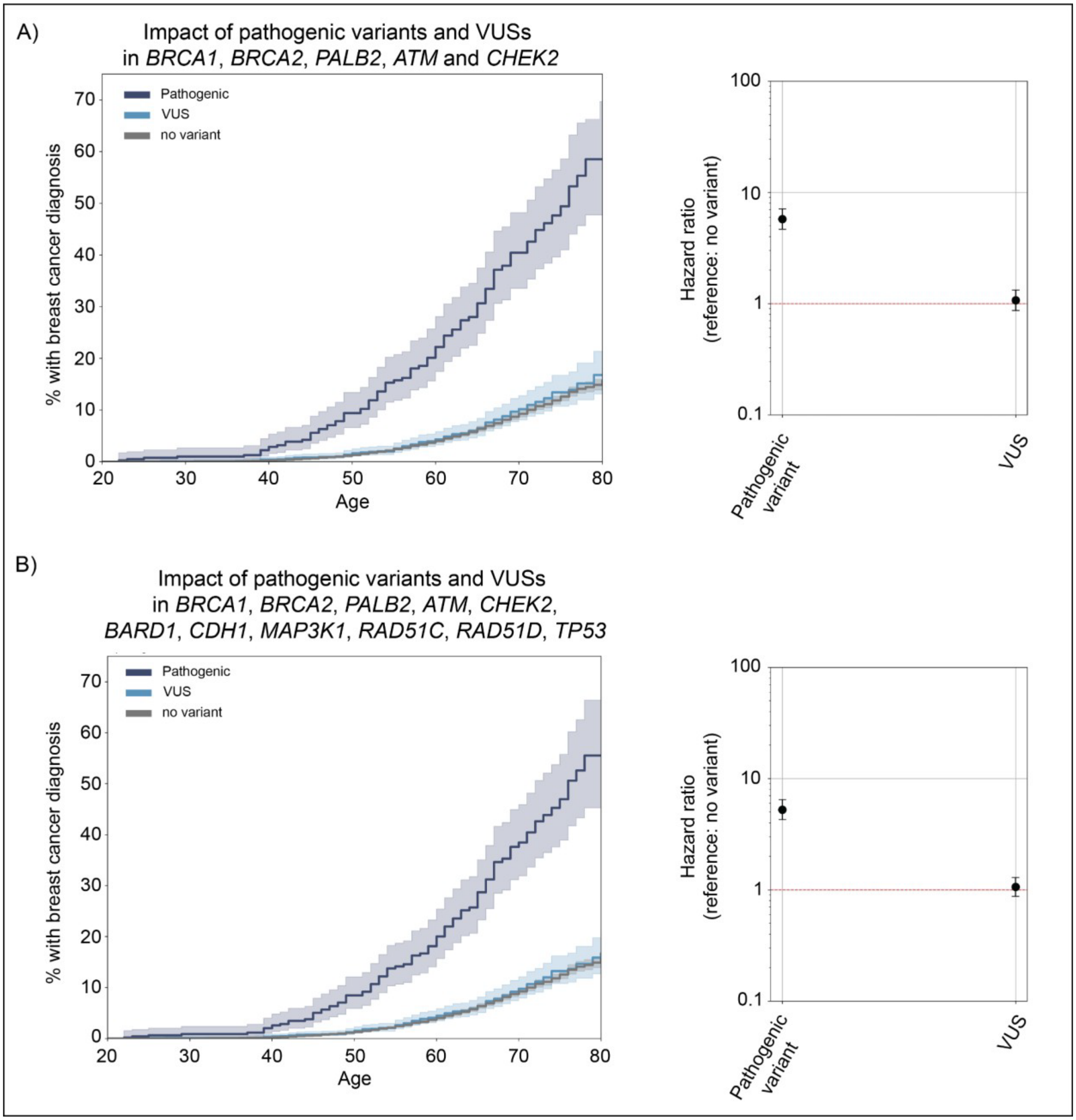

eFigure 2: Impact of pathogenic variants and VUSs on breast cancer diagnosis

(A) Analysis for the 5 genes: *BRCA1*, *BRCA2*, *PALB2*, *ATM* and *CHEK2*. Left panel: Kaplan Meier curves showing the % of women with a breast cancer diagnosis by age based on whether they have: a pathogenic variant in one of the 5 genes (dark blue curve), a VUS in one of the 5 genes (light blue curve), or no P or VUS variant in these 5 genes (gray curve). 95% Confidence intervals are represented in lighter shades. Right panel: Cox proportional hazard ratios and their

© 2023 American Medical Association. All rights reserved.

95% Confidence intervals; Y-axis is on a log scale. Reference group is the group without a P or VUS variant in the 5 genes. **(B)** Analysis for the 11 genes: *BRCA1*, *BRCA2*, *PALB2*, *ATM*, *CHEK2*, *BARD1*, *CDH1*, *MAP3K1*, *RAD51C*, *RAD51D*, and *TP53*. Left panel: Kaplan Meier curves showing the % of women with a breast cancer diagnosis by age based on whether they have: a pathogenic variant in one of the 11 genes (dark blue curve), a VUS in one of the 11 genes (light blue curve), or no P or VUS variant in these 11 genes (gray curve). 95% Confidence intervals are represented in lighter shades. Right panel: Cox proportional hazard ratios and their 95% Confidence intervals; Y-axis is on a log scale. Reference group is the group without a P or VUS variant in the 11 genes.

© 2023 American Medical Association. All rights reserved.

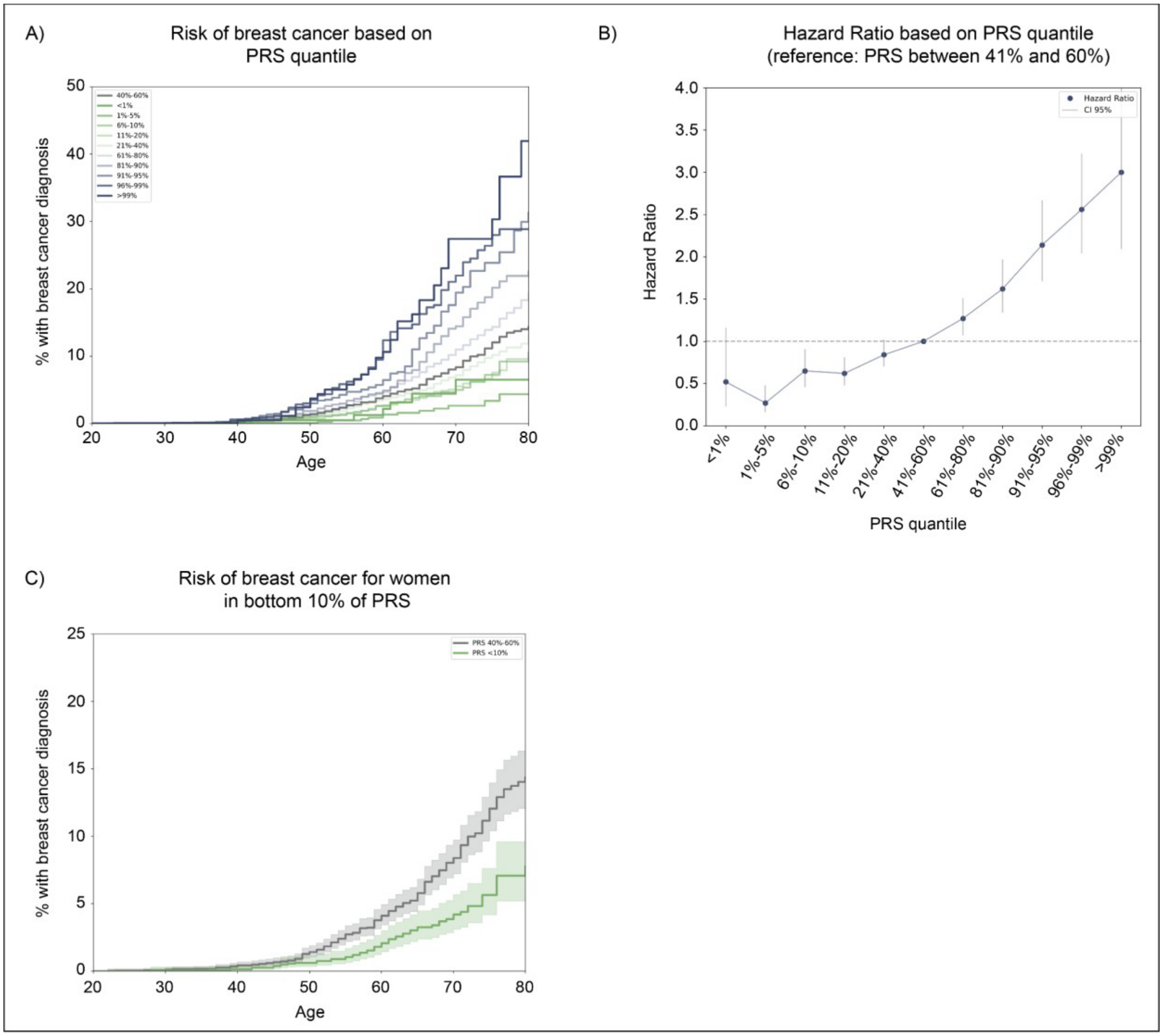

eFigure 3: Impact of polygenic risk on breast cancer diagnosis

(A) Kaplan Meier curves showing the % of women with a breast cancer diagnosis by age based on their polygenic risk quantile. Blue shades indicate higher quantiles (higher polygenic risk). Green shades indicate lower quantiles (lower polygenic risk). Gray curve represents the average risk for women with a PRS between 41% and 60% of the PRS distribution. **(B)** On the Y-axis: Cox proportional hazard ratios and their 95% Confidence intervals calculated for each PRS group compared to the reference group which is the 41%-60% group. **(C)** Kaplan Meier curves showing the % of women with a breast cancer diagnosis by age based on their polygenic risk. Green curve represents women with a low polygenic risk (the combined groups: <1%, 1%-

© 2023 American Medical Association. All rights reserved.

5% and 6%-10%). Gray curve represents the average risk for women with a PRS between 41% and 60% of the PRS distribution. 95% Confidence intervals are represented in lighter shades.

© 2023 American Medical Association. All rights reserved.

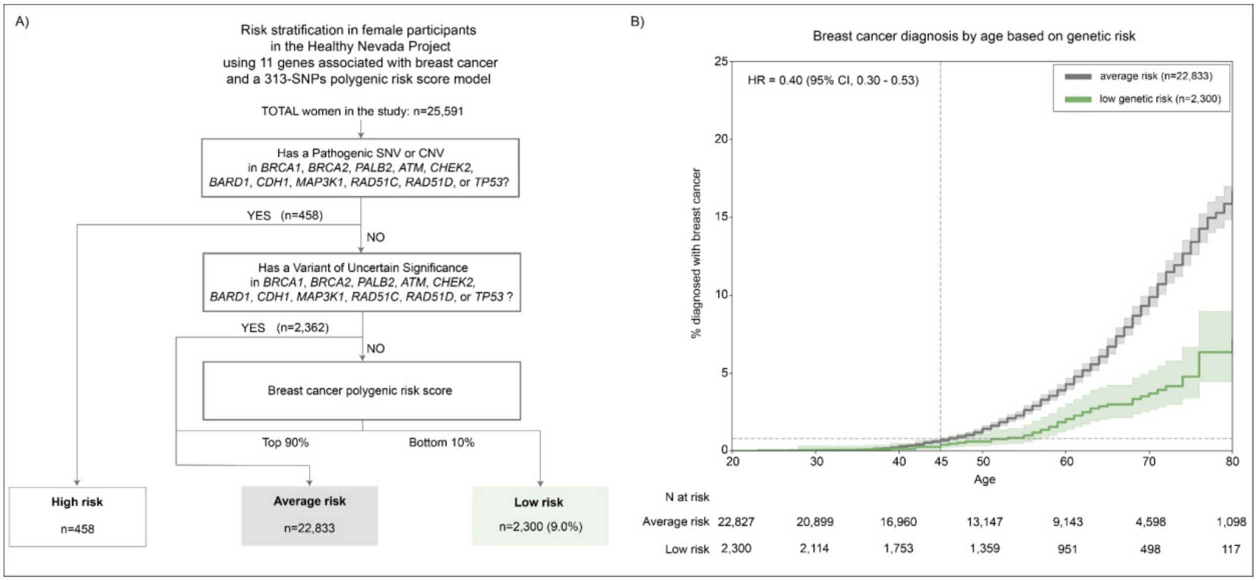

eFigure 4: Impact of low genetic risk based on 11 genes and a 313-SNPs PRS on breast cancer diagnosis

(A) Schema detailing how genetic risk stratification was done. **(B)** Kaplan Meier curves showing the % of women with a breast cancer diagnosis by age based on their genetic risk group: average risk (gray curve), low risk (green curve). 95% confidence intervals are represented in light gray and light green. The KaplanMeierFitter function (to draw the curves) and the CoxPHFitter function (to calculate the Hazard Ratio) were used and were from the Lifelines python library.

© 2023 American Medical Association. All rights reserved.

Supplementary Tables

eTable 1: Description of the Healthy Nevada Project

eTable 2: List of all Pathogenic variants in 11 breast cancer genes

eTable 3: List of all Pathogenic CNVs in 11 breast cancer genes

eTable 4: List of all VUSs in 11 breast cancer genes

eTable 5: Association of the 313-SNPs breast cancer PRS and breast cancer in the Healthy Nevada Project cohort

eTable 6: Impact of delay of screening for those in the low-risk group defined using 11 genes and a PRS

© 2023 American Medical Association. All rights reserved.

eReferences

1. Venkatesan P. New US breast cancer screening recommendations. *Lancet Oncol*. Published online May 18, 2023. doi:10.1016/S1470-2045(23)00238-3
2. US Census Bureau. Age and sex composition in the United States: 2022. Published online August 29, 2023. Accessed August 31, 2023. https://www.census.gov/data/tables/2022/demo/age-and-sex/2022-age-sex-composition.html
3. Lowry KP, Geuzinge HA, Stout NK, et al. Breast Cancer Screening Strategies for Women With ATM, CHEK2, and PALB2 Pathogenic Variants: A Comparative Modeling Analysis. *JAMA Oncol*. 2022;8(4):587-596. doi:10.1001/jamaoncol.2021.6204
4. Manahan ER, Kuerer HM, Sebastian M, et al. Consensus Guidelines on Genetic` Testing for Hereditary Breast Cancer from the American Society of Breast Surgeons. *Ann Surg Oncol*. 2019;26(10):3025-3031. doi:10.1245/s10434-019-07549-8
5. Lee A, Mavaddat N, Cunningham A, et al. Enhancing the BOADICEA cancer risk prediction model to incorporate new data on RAD51C, RAD51D, BARD1 updates to tumour pathology and cancer incidence. *J Med Genet*. 2022;59(12):1206-1218. doi:10.1136/jmedgenet-2022-108471
6. Kuchenbaecker KB, Hopper JL, Barnes DR, et al. Risks of Breast, Ovarian, and Contralateral Breast Cancer for BRCA1 and BRCA2 Mutation Carriers. *JAMA*. 2017;317(23):2402-2416. doi:10.1001/jama.2017.7112
7. Wilcox N, Dumont M, González-Neira A, et al. Exome sequencing identifies breast cancer susceptibility genes and defines the contribution of coding variants to breast cancer risk. *Nat Genet*. Published online August 17, 2023. doi:10.1038/s41588-023-01466-z
8. Bolze A, Kiser D, Schiabor Barrett KM, et al. Combining rare and common genetic variants improves population risk stratification for breast cancer. *medRxiv*. Published online May 19, 2023. doi:10.1101/2023.05.17.23290132
9. Easton DF, Pharoah PDP, Antoniou AC, et al. Gene-panel sequencing and the prediction of breast-cancer risk. *N Engl J Med*. 2015;372(23):2243-2257. doi:10.1056/NEJMsr1501341
10. Mavaddat N, Michailidou K, Dennis J, et al. Polygenic Risk Scores for Prediction of Breast Cancer and Breast Cancer Subtypes. *Am J Hum Genet*. 2019;104(1):21-34. doi:10.1016/j.ajhg.2018.11.002
11. Grzymski JJ, Elhanan G, Morales Rosado JA, et al. Population genetic screening efficiently identifies carriers of autosomal dominant diseases. *Nat Med*. 2020;26(8):1235-1239. doi:10.1038/s41591-020-0982-5
12. Harrison SM, Rehm HL. Is “likely pathogenic” really 90% likely? Reclassification data in ClinVar. *Genome Med*. 2019;11(1):72. doi:10.1186/s13073-019-0688-9

© 2023 American Medical Association. All rights reserved.

13. Maas P, Barrdahl M, Joshi AD, et al. Breast Cancer Risk From Modifiable and Nonmodifiable Risk Factors Among White Women in the United States. *JAMA Oncol*. 2016;2(10):1295-1302. doi:10.1001/jamaoncol.2016.1025

14. Kachuri L, Chatterjee N, Hirbo J, et al. Principles and methods for transferring polygenic risk scores across global populations. *Nat Rev Genet*. Published online August 24, 2023. doi:10.1038/s41576-023-00637-2

15. Zhang H, Ahearn TU, Lecarpentier J, et al. Genome-wide association study identifies 32 novel breast cancer susceptibility loci from overall and subtype-specific analyses. *Nat Genet*. 2020;52(6):572-581. doi:10.1038/s41588-020-0609-2

16. McLaren W, Gil L, Hunt SE, et al. The Ensembl Variant Effect Predictor. *Genome Biol*. 2016;17(1):122. doi:10.1186/s13059-016-0974-4

17. Karczewski KJ, Francioli LC, Tiao G, et al. The mutational constraint spectrum quantified from variation in 141,456 humans. *Nature*. 2020;581(7809):434-443. doi:10.1038/s41586-020-2308-7

18. Morales J, Pujar S, Loveland JE, et al. A joint NCBI and EMBL-EBI transcript set for clinical genomics and research. *Nature*. 2022;604(7905):310-315. doi:10.1038/s41586-022-04558-8

19. Lee A, Mavaddat N, Wilcox AN, et al. BOADICEA: a comprehensive breast cancer risk prediction model incorporating genetic and nongenetic risk factors. *Genet Med*. 2019;21(8):1708-1718. doi:10.1038/s41436-018-0406-9

20. Breast Cancer Association Consortium, Dorling L, Carvalho S, et al. Breast Cancer Risk Genes - Association Analysis in More than 113,000 Women. *N Engl J Med*. 2021;384(5):428-439. doi:10.1056/NEJMoa1913948

21. Shahbandi A, Nguyen HD, Jackson JG. TP53 Mutations and Outcomes in Breast Cancer: Reading beyond the Headlines. *Trends Cancer Res*. 2020;6(2):98-110. doi:10.1016/j.trecan.2020.01.007

22. Corso G, Veronesi P, Sacchini V, Galimberti V. Prognosis and outcome in CDH1-mutant lobular breast cancer. *Eur J Cancer Prev*. 2018;27(3):237-238. doi:10.1097/CEJ.0000000000000405

© 2023 American Medical Association. All rights reserved.
