# Supplementary material for: The Potential of Genetics in Identifying Women at Lower Risk of Breast Cancer
Source: JAMA Oncol. 2023 Dec 28;10(2):236–9. doi: 10.1001/jamaoncol.2023.5468 (PMC10870185; doi:10.1001/jamaoncol.2023.5468)
Supplement: Supplement 3. — Data Sharing Statement [file jamaoncol-e235468-s003.pdf]

Data Sharing Statement

Bolze. The Potential of Genetics in Identifying Women at Lower Risk of Breast Cancer. *JAMA Oncol*. Published December 28, 2023. doi:10.1001/jamaoncol.2023.5468

Data
Data available: Yes
Data types: Deidentified participant data
How to access data: A table with the data used to create Table 1 and Figure 1 of the manuscript is uploaded on Mendeley data. It has one row per individual so 25,591 rows. There is a DOI associated with it: http://dx.doi.org/10.17632/n6c36j4fn2.1
When available: With publication

Supporting Documents
Document types: Statistical/analytic code
How to access documents: The notebooks and code used for the statistical analyses will be uploaded to Mendeley data just like the individual de-identified data.
When available: With publication

Additional Information
Who can access the data: The data will be available to anyone.
Types of analyses: The data can be used to replicate the figure and table in the manuscript. It could be used to look at the impact of different screening strategies not discussed in the paper. Overall, analyses looking at KaplanMeier curves and screening performance can be done with the data shared.
Mechanisms of data availability: Via Mendeley Data. A table with the data has already been deposited.
